# Traffic-related exposures and biomarkers of systemic inflammation, endothelial activation and oxidative stress: a panel study in the US trucking industry

**DOI:** 10.1186/1476-069X-12-105

**Published:** 2013-12-07

**Authors:** Andreas M Neophytou, Jaime E Hart, Jennifer M Cavallari, Thomas J Smith, Douglas W Dockery, Brent A Coull, Eric Garshick, Francine Laden

**Affiliations:** 1 Exposure Epidemiology and Risk Program, Department of Environmental Health, Harvard School of Public Heath, Boston, MA, USA; 2 Channing Division of Network Medicine, Brigham and Women’s Hospital and Harvard Medical School, Boston, MA, USA; 3 Division of Occupational and Environmental Medicine, University of Connecticut Health Center, Farmington, CT, USA; 4 Department of Biostatistics, Harvard School of Public Health, Boston, MA, USA; 5 VA Boston Healthcare System, West Roxbury, MA, USA

**Keywords:** Particulate matter, Elemental carbon, Organic carbon, ICAM-1, VCAM-1, IL-6, CRP, Inflammation, Endothelial activation, Trucking industry

## Abstract

**Background:**

Experimental evidence suggests that inhaled particles from vehicle exhaust have systemic effects on inflammation, endothelial activation and oxidative stress. In the present study we assess the relationships of short-term exposures with inflammatory endothelial activation and oxidative stress biomarker levels in a population of trucking industry workers.

**Methods:**

Blood and urine samples were collected pre and post-shift, at the beginning and end of a workweek from 67 male non-smoking US trucking industry workers. Concurrent measurements of microenvironment concentrations of elemental and organic carbon (EC & OC), and fine particulate matter (PM_2.5_) combined with time activity patterns allowed for calculation of individual exposures. Associations between daily and first and last-day average levels of exposures and repeated measures of intercellular and vascular cell adhesion molecule-1 (ICAM-1 & VCAM-1), interleukin 6 (IL-6) and C-reactive protein (CRP) blood levels and urinary 8-Hydroxy-2′-Deoxyguanosine (8-OHdG) were assessed using linear mixed effects models for repeated measures.

**Results:**

There was a statistically significant association between first and last-day average PM_2.5_ and 8-OHdG (21% increase, 95% CI: 2, 42%) and first and last-day average OC and IL-6 levels (18% increase 95% CI: 1, 37%) per IQR in exposure. There were no significant findings associated with EC or associations suggesting acute cross-shift effects.

**Conclusion:**

Our findings suggest associations between weekly average exposures of PM_2.5_ on markers of oxidative stress and OC on IL-6 levels.

## Background

Vehicle exhaust related particulate air pollution has been associated with cardiovascular health outcomes, including, ventricular arrhythmias, onset of myocardial infarction and increased cardiovascular mortality [1-7], while occupational exposures to vehicle exhaust also have been shown to be associated with ischemic heart disease [8,9]. We have previously observed an increased risk of ischemic heart disease mortality in US trucking industry workers with regular exposures to exhaust from diesel, gasoline and propane source [10]. Occupational air pollution exposures in the trucking industry are relevant to the general population due to overlap with levels experienced during daily activities such as commuting [11,12].

Potential pathways for these outcomes include systemic inflammation, endothelial dysfunction and oxidative stress, which experimental animal and human studies suggest are associated with traffic-related particulate matter (PM) [13-20]. Associations between markers of cellular damage and induction of inflammatory responses and oxidative stress, however, are not adequately defined and have not been well characterized in a population with vehicle exhaust exposures in a non-experimental setting.

Previous studies have shown associations between biomarkers of endothelial function such as transmembrane proteins intercellular adhesion molecule-1 (ICAM-1) and vascular cell adhesion molecule-1 (VCAM-1), and air pollution exposures [21]. ICAM-1 and VCAM-1 levels are associated with increased cardiovascular risk and are predictors of acute coronary events [22-24]. General markers of inflammation like interleukin-6 (IL-6) and C-reactive protein (CRP) have also been associated with cardiovascular risk [25,26] and air pollution [27,28]. In addition, CRP has recently been linked to cancer mortality in general and lung cancer incidence in particular [29,30]. Urinary 8-Hydroxy-2′-Deoxyguanosine (8-OHdg) is a DNA lesion repair product [31], suitable as an oxidative damage biomarker that has also been shown to be associated with particulate exposures [15,32,33].

The current study characterizes exposures to different components of engine exhaust in a sample of workers in the trucking industry and examines the relation between exposure and inflammation, endothelial activation, and oxidative stress as indicated by plasma levels of IL-6, CRP, ICAM-1 and VCAM-1 and by urinary levels of 8-OHdG from repeated blood and urine samples, sampled over the duration of one work-week. The study examines associations over different time-windows for both acute and more intermediate-term effects.

## Methods

### Study population

We studied a sample of 95 workers originated from 10 work locations (truck terminals) in the northeastern US (specifically CT, MA, MD, NJ, NY, and PA). Primary analyses were restricted to non-smoking males with biological samples who did not report sick days during the sampling period (n = 67). Measurements took place between February 2009 and October 2010, with terminals sampled one at time for up to 8 days of continuous sampling. Subjects were selected from 3 different job categories representing different exposure scenarios: pick-up and delivery (P&D) drivers, freight dockworkers and office clerks. Participants provided informed consent and were compensated at the end of the protocol for their participation. The protocol was approved by the Institutional Review Board of the Brigham and Women’s Hospital and the Human Subjects Committee of the Harvard School of Public Health.

### Microenvironment exposure measurements

Microenvironment area samples of PM_2.5_ (particulate matter with a diameter of ≤2.5 μm)_,_ as well as elemental carbon (EC) and organic carbon (OC) in PM_1.0_ (particulate matter with a diameter of ≤1.0 μm) were collected from all 10 terminals in the study. Twelve-hour integrative area samples were collected indoors in office spaces and terminal docks 24 hours a day. For the P&D drivers, samplers were placed in their truck cabs for the duration of each work-shift on the days of biological sample collection. Detailed exposure assessment for each of the three pollutants is described elsewhere [11]. Briefly EC and OC were measured by collecting PM_1.0_ on a 22-mm quartz tissue filter, preceded by a precision machined cyclone separator (SCC1.062 Triplex, BGI, Inc., Waltham, MA), which was then analyzed by thermal-optical carbon analyzer using the NIOSH 5040 method [34]. PM_2.5_ was collected on a 37-mm Teflon filter (with a pore diameter of 2.0 μm) after passing through a precision-machined cyclone pre-selector to remove particles greater than 2.5 μm aerodynamic diameter. The method was consistent with the EPA PQ200 Federal Reference Method [35,36]. A flow rate of 3.5 liters per minute was used for both PM_2.5_ and PM_1.0_ samples. Exposures were assigned to each individual participant as a weighted average of the time spent in each work location based on the samples in each location during each work shift.

### Biomarker sampling

During each truck terminal visit, initial blood samples were drawn and urine was collected from study participants prior to the day’s work shift on their first day back to work after at least 2 days off. At the end of the first work shift a second blood and urine sample were collected and then pre- and post-shift samples were collected again on the last workday of the same week. At each blood draw, two 10-ml liquid EDTA blood tubes were drawn and stored at 4°C until shipped in an insulated container to our blood processing laboratory where they were centrifuged, aliquoted and stored in the vapor phase of liquid nitrogen freezers at < -130°C. Up to 50 ml of urine were collected in a sterile urine cup at each sampling period and stored at 4°C until returned to the study laboratory, where they were kept at -20°C until analysis.

Analysis of CRP, IL-6, ICAM-1 and VCAM-1 was done at the Clinical and Epidemiologic Research Laboratory, Department of Laboratory Medicine at Children’s Hospital in Boston, a state-of the-art laboratory specializing in micro-analysis. The stability of the selected biomarkers was assessed by processing at 0, 24 and 36 hours after packaging and storage on ice in a shipping container and there were no differences in the measured marker levels [37].

CRP concentrations were determined with the use of a high sensitivity immonoturbidimetric assay, with a sensitivity of 0.03 mg/L. Values of CRP > 10 mg/L (n = 3) were excluded from the analysis as they suggest elevation due to recent acute illness. IL-6, ICAM-1 and VCAM-1 were measured using ELISA assays with sensitivities of 0.094 pg/mL, 0.35 ng/mL and 0.6 ng/mL, respectively. Urinary 8-OHdG levels were measured in the laboratory of Dr. Junfeng Zhang at the University of Southern California using a high performance liquid chromatography with electrochemical detection (HPLC-ECD) method with a level of detection (LOD) of 0.46 ng/mL. Urinary creatinine was also measured and 8-OHdG levels were corrected and reported as μg/g creatinine. Three subjects contributed no or insufficient blood sample volumes for analysis on at least one measurement occasion and analysis was restricted to the remaining occasions for these individuals. None of the analyzed samples were below the stated LODs.

### Health and personal habit assessment

A baseline medical and health questionnaire based on the American Thoracic Society adult respiratory questionnaire [38] was administered to all participants. The questionnaire included standardized questions regarding respiratory symptoms and conditions, and was supplemented with questions about other medical conditions such as heart disease, cancer, and diabetes. Information was also provided on job title and job history, date and time of last work shift, past week work schedule, terminal assignment, specific duties, recent acute illnesses, and lifestyle characteristics such as physical activity and smoking history. A personal habits portion of the questionnaire including medication use (such as aspirin, blood pressure medications and lipid lowering agents), smoking, and second hand smoke (SHS) exposure as well as information on job duties during the day, and timing and location of all breaks, was administered at the end of each shift during the work week. Weight and height were measured by the study team to calculate body mass index (BMI).

### Statistical analyses

Marker levels were natural log-transformed and proportional changes are reported. Linear mixed effects models for repeated measures with unstructured autocorellation, were used to estimate the effects each of the measured pollutants on marker levels for all study participants in the primary analysis study population. We included a random intercept for each participant to account for baseline inter-individual differences. Personal characteristics including known predictors of CRP, IL-6, ICAM-1 and VCAM-1 such as age, BMI, physician diagnosed hypertension, physical activity, chronic disease, and medication use were included as confounders *a priori* in the models. Models for 8-OHdG included age, BMI, physical activity, diabetes, and chronic respiratory conditions, which are thought to be associated with oxidative damage [39,40]. Additional covariates such as terminal (accounting for possible seasonal and regional differences), past smoking history, self reported SHS exposures during the work shift, and switching shifts were also considered as potential confounders and were kept in the models if they changed the primary effect estimate by ≥10%. The models fitted were as follows:

$$\begin{array}{ll} Y_{ijk}=\beta_{0} & +\beta_{1}{\left(\text{Exp}\right)}_{i}+\beta_{2}{\left(\text{day}\right)}_{j}+\beta_{3}{\left(\text{shift}\right)}_{k}\\ & +\beta_{4}{\left(\text{day}*\text{shift}\right)}_{jk}+\gamma {\left(\mathrm{cov}\text{ariates}\right)}_{ijk}\\ & +b_{i}+\epsilon_{\text{ijk}} \end{array}$$

where:

• Y_ijk_: log-transformed marker levels for subject i, on day j, at the k (pre-or post-shift) measurement point

• Exp_i_: Entered as the first and last-day average of the daily pollutant levels on the two measured work-shifts

• Day_j_: Measurement day j: first or last day worked during the week

• Shift_k_: Measurement time k relevant to shift: pre- or post-shift

• Covariates_ijk_: Covariates entered in the model depending on biomarker of interest as listed above.

• b_i_: random intercept for each individual i

• ϵ_ijk_: within subject error

The average exposure model was fitted to examine possible effects that persist rather than being acute. The day and shift variables and their interaction allow outcome measurements to vary with time irrespective of exposure or covariate values. To examine for any transient effects the following model was fitted:

$$\begin{array}{ll} Y_{\mathrm{ij}}=\beta_{0} & +\beta_{1}{\left(\mathrm{Exp}\right)}_{\mathrm{ij}}+\beta_{2}{\left(\mathrm{day}\right)}_{j}+\gamma {\left(\text{cov}\mathrm{ariates}\right)}_{\mathrm{ij}}\\ & +b_{i}+\epsilon_{\mathrm{ij}} \end{array}$$

Using only post-shift measurements where Exp_ij_ is the estimated daily exposure for each participant on a given day, and the other variables are as described above.

In the case of statistically significant findings, multi-pollutant models were fitted. Models with penalized spline terms instead of linear terms for exposures were fitted to examine the shape of dose-responses. Finally, exposure time (day and shift) interactions were also included and the choice of the final model was decided by likelihood ratio tests. Statistical analysis was performed using the SAS mixed procedure (SAS version 9.3; SAS Institute Inc., Cary, NC) and penalized spline models were fitted using the R mgcv package (R, version 2.15.2).

## Results

### Study population

Descriptive statistics for the participants in the primary analysis are presented in Table 1 collectively and by job title. Briefly, the study sample was predominantly white (93%), while the age range was 23 to 66 years, with a mean ± standard deviation (SD) of 50.4 ± 8.5. The range for the length of the workweek among study participants was 2 to 5 days with a mean of 4 days. The range of shift length was 2.2 to 13.4 hours with a mean of 9.5 hours per shift and an average of 45.8 hours worked during the week, with drivers working longer hours that the other two job groups. Overall this was a healthy working population except for elevated BMI levels (mean BMI was 30.4 ± 4.4), with few reports of chronic disease. Twenty-four (36%) participants reported use of lipid lowering agents and nineteen (28%) reported use of blood pressure medication. Study participants appeared to be similar across job titles with the exceptions that P&D Drivers were more likely to be ex-smokers. One-Way Analysis of Variance indicated significant differences across job title levels in only the means of length of work week and total hours worked out of all covariates presented (p-value for F-test <0.01 for both).

**Table 1:** Study population baseline characteristics in a sample of male, non-smoking trucking industry workers

	**Total**	**P&D driver**	**Dock**	**Office**
Total no.	67	35	14	18
Race (no. (%))				
White	62 (93%)	32 (91%)	14 (100%)	16 (89%)
Hispanic	5 (7%)	3 (9%)	0 (0%)	2 (11%)
Age (years, mean ± SD)	50.4 ± 8.5	51.1 ± 7.8	47.9 ± 11.0	50.8 ± 7.5
BMI (kg/m^2^, mean ± SD)	30.4 ± 4.4	30.8 ± 4.1	29.8 ± 4.3	30.1 ± 5.2
Past smoker (no. (%))	36 (54%)	23 (66%)	6 (43%)	7 (39%)
Exercise (hrs/wk, mean ± SD)	3.3 ± 3.7	2.3 ± 2.9	4.6 ± 5.4	4.4 ± 2.7
Hypertension (no. (%))	24 (36%)	13 (37%)	6 (43%)	5 (28%)
Heart disease (no. (%))	3 (4%)	0 (0%)	1 (7%)	2 (11%)
CHD (no. (%))	6 (9%)	4 (11%)	1 (7%)	1 (6%)
Diabetes (no. (%))	1 (1.5%)	1 (3%)	0 (0%)	0 (0%)
Medication use				
Blood pressure	19 (28%)	9 (26%)	5 (36%)	5 (28%)
Lipid lowering	24 (36%)	14 (40%)	4 (29%)	6 (33%)
Aspirin	17 (25%)	9 (26%)	2 (14%)	6 (33%)
Total workdays (mean ± SD)	4.0 ± 0.5	3.9 ± 0.4	4.4 ± 0.5	3.9 ± 0.5
Avg shift duration (hrs, mean ± SD)	9.5 ± 1.8	10.6 ± 1.0	8.6 ± 1.6	8.1 ± 1.9
Total hrs worked during study period	45.8 ± 9.4	50.7 ± 6.9	41.7 ± 9.3	39.3 ± 8.8

### Exposure measurements

**Figure 1:**
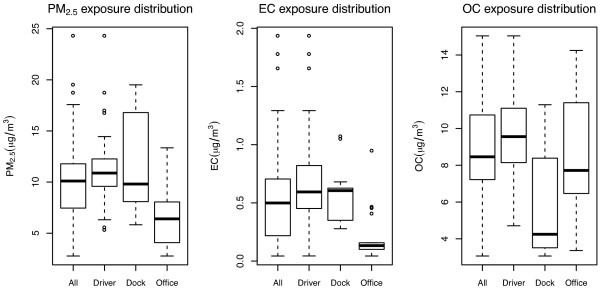
**Boxplots for first and last-day average exposure levels.** PM_2.5_, EC, OC levels by job title and all job-titles combined. The outer box lines represent the first and third quartiles, while the thick horizontal line in the box represents the median. The “whiskers” extend to the values of the sample within 1.5 IQR of the lower and upper quartiles, and values outside this range are represented as circles.

Estimated individual exposure levels are presented in Figure 1. Overall first-and-last-day average (± SD) levels for the three pollutants examined were 10.22 (± 4.30) μg/m^3^ for PM_2.5_, 0.54 (± 0.40) μg/m^3^ for EC and 8.60 (± 2.91) μg/m^3^ for OC. Dockworkers and P&D drivers had higher PM_2.5_ and EC exposures compared to office clerks, while OC was highest among P&D drivers followed by office clerks. Correlations between average exposures were not high with pearson coefficients of 0.370 between PM_2.5_ and EC, 0.344 between PM_2.5_ and EC, and 0.004 between EC and OC. One-Way Analysis of Variance indicated significant differences for the means all three pollutants across the different job title levels (p-value for F-test <0.001 for all three pollutants).

### Associations between biomarker levels and exposures

**Figure 2:**
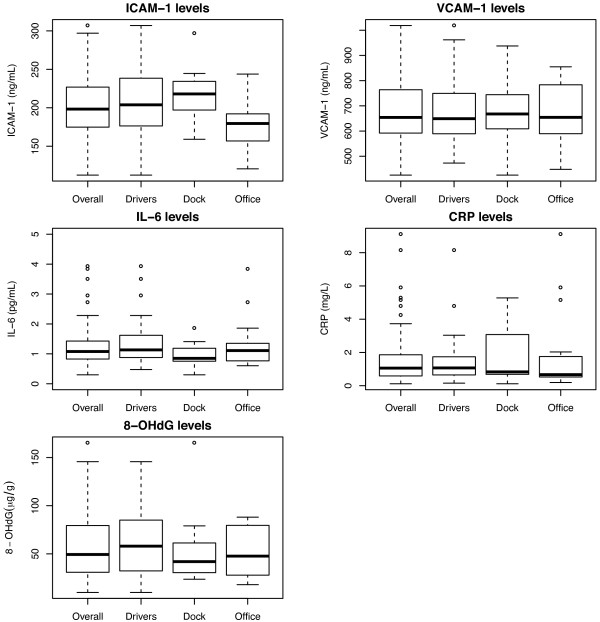
**Boxplots for endothelial activation, inflammation, and oxidative stress biomarker levels.** Average levels from the four measurement occasions used for each participant, shown for by job title and all job-titles combined.

From the 67 participants, a total of 264 blood (ICAM-1, VCAM-1, IL-6, CRP) and 268 urine samples (8-OHdG) were analyzed. The distributions of subject specific mean biomarker levels are shown in Figure 2, while mean (± SD), medians, geometric means (GM) and geometric standard deviations (GSD) are summarized in Table 2 (Presented here as the average of four collection time points for descriptive purposes; biomarker measurements were entered individually in all models considered). For the endothelial activation markers, there was little difference in the means by job title for VCAM-1, but mean ICAM-1 levels were higher among P&D drivers and dockworkers compared to office clerks (p-value for One-Way Analysis of Variance F-Test, p = 0.03). However, neither ICAM-1 nor VCAM-1, were associated with PM_2.5_, EC, and OC levels for either the average (Table 3) or daily shift exposures (Table 4).

**Table 2:** Mean (± SD) and geometric mean (± GSD) for subject specific mean marker levels averaged over the 4 different measurement points

**Biomarker**	**Overall**		**P&D drivers**		**Dockworkers**		**Office clerks**	
	**Mean (±SD)**	**GM (±GSD)**	**Mean (±SD)**	**GM (±GSD)**	**Mean (±SD)**	**GM (±GSD)**	**Mean (±SD)**	**GM (±GSD)**
ICAM-1 (ng/mL)	202 ± 40	198 ± 1.22	208 ± 42	203 ± 1.23	216 ± 34	213 ± 1.17	181 ± 32	178 ± 1.19
VCAM-1 (ng/mL)	677 ± 131	661 ± 1.21	679 ± 144	663 ± 1.23	673 ± 123	658 ± 1.21	675 ± 115	661 ± 1.19
IL-6 (pg/mL)	1.38 ± 1.20	1.09 ± 1.71	1.36 ± 0.79	1.12 ± 1.59	1.50 ± 2.17	0.98 ± 2.14	1.30 ± 0.82	1.11 ± 1.61
CRP (mg/L)	1.66 ± 1.80	1.00 ± 2.63	1.53 ± 1.51	1.01 ± 2.36	1.72 ± 1.68	0.98 ± 3.18	1.87 ± 2.41	1.01 ± 2.90
8-OHdG (μg/g creat.)	55.9 ± 31.5	47.7 ± 1.79	58.9 ± 32.9	49.3 ± 1.90	52.8 ± 36.1	45.8 ± 1.67	52.3 ± 25.4	46.0 ± 1.72

**Table 3:** **Percent change and 95**% **confidence intervals in biomarker levels associated with each IQR increase in average exposure levels**^**a**^

**Exposure**^**b**^	**IQR**	**ICAM-1**	**VCAM-1**	**IL-6**	**CRP**	**8-OHdG**
PM_2.5_	4.59	3.5 (-1.1, 8.4)	0.4 (-4.3, 5.4)	3.3 (-8.6, 16.7)	-6.3 (-25.1, 17.3)	20.6 (2.4, 42.0)
EC	0.51	-1.2 (-6.8, 4.6)	-1.7 (-7.4, 4.3)	-3.1 (-16.7, 12.8)	-15.0 (-35.6, 12.2)	17.5 (-4.6, 44.7)
OC	3.62	-2.2 (-7.8, 3.8)	2.4 (-3.6, 8.9)	17.7 (1.2, 36.9)	4.1 (-21.9, 38.8)	10.4 (-11.3, 37.4)

Notes: ^a^Linear mixed effect models for repeated measures using all 4 measurements per participant, with random intercepts for each individual and adjusting for age, BMI, physical activity, hypertension, chronic disease, SHS exposure and in the case of inflammation and endothelial activation markers time varying medication use.

^b^Pollutant models with first and last-day average exposure values (average of available daily exposures over the work-week) as the exposure of interest, with each pollutant modelled separately.

**Table 4:** **Percent change and 95% confidence intervals in biomarker levels associated with each IQR increase in daily exposure levels**^**a**^

**Exposure**^**b**^	**IQR**	**ICAM-1**	**VCAM-1**	**IL-6**	**CRP**	**8-OHdG**
PM_2.5_	6.11	-1.1 (-3.7, 1.5)	-1.8 (-4.2, 0.7)	2.2 (-7.6, 13.0)	-0.2 (-13.1, 14.8)	8.9 (-6.5, 26.5)
EC	0.56	0.1 (-2.5, 2.8)	-0.3 (-2.8, 2.3)	-7.1 (-14.6, 1.0)	-5.3 (-13.7, 4.0)	4.9 (-9.3, 21.3)
OC	3.97	-1.1 (-4.4, 2.2)	-1.1 (-4.3, 2.1)	12.2 (-0.4, 25.8)	1.2 (-14.7, 20.0)	-3.1 (-18.7, 15.4)

Notes: ^a^Linear mixed effect models for repeated measures using only the 2 post-shift measurements per participant, with random intercepts for each individual and adjusting for age, BMI, physical activity, hypertension, chronic disease, SHS exposure and in the case of inflammation and endothelial activation markers time varying medication use.

^b^Pollutant models with daily exposure values as the exposure of interest, with each pollutant modelled separately.

Little difference was seen in inflammation (CRP, IL-6) biomarker levels across job titles (Table 2). No associations with any of the pollutant exposures were seen for CRP, but a statistically significant association between IL-6 and first and last-day average OC levels was observed (17.7% increase per IQR in exposure, 95% CI: 1.2, 36.9%) (Table 3). The OC effect size (18.7%, 95% CI: 0.7, 39.8%) was stable in the multi-pollutant model, where first and last-day average PM_2.5_ and EC were included at the same time (Table 5). No significant associations were observed between any of the daily exposures and inflammation marker levels (Table 4).

**Table 5:** **Percent change and 95% confidence intervals of IL-6 and 8-OHdG levels associated in multi-exposure models of first and last-day average exposures**^**a**^

**Exposure**^**b**^	**IL-6**	**8-OHdG**
PM_2.5_	-0.6 (-13.4, 14.0)	16.7 (-3.3, 40.9)
EC	-4.4 (-18.3, 11.9)	9.1 (-12.4, 36.0)
OC	18.7 (0.7, 39.8)	2.2 (-18.5, 28.2)

Notes: ^a^Linear mixed effect models for repeated measures using all 4 measurements per participant, with random intercepts for each individual and adjusting for age, BMI, physical activity, hypertension, chronic disease, SHS exposure and in the case of IL-6 time varying medication use.

^b^Models included first and last-day-average values for PM_2.5_, EC and OC in the same model.

Mean 8-OHdG levels were similar among all job titles (Table 2). However, there was a statistically significant increase in 8-OHdG levels associated with each IQR increase in first and last-day average PM_2.5_ (20.6%, 95% CI: 2.4, 42.0%) (Table 3). The PM_2.5_ effect size (16.7%, 95% CI: -3.3, 40.9%) remained elevated though not statistically significant in the model that included all three pollutants (Table 5). Again, no significant associations were observed between any of the daily exposures and 8-OHdG (Table 4).

**Figure 3:**
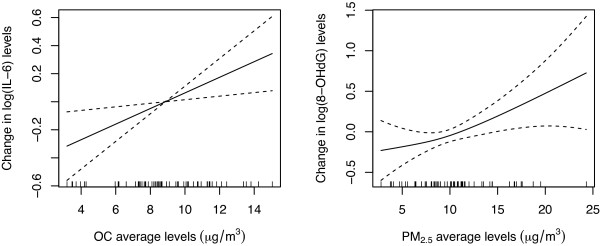
**Exposure-response penalized spline term graphs.** Associations between OC first and last-day average levels and IL-6 (left, effective degrees of freedom (edf) = 1, p = 0.01) and PM_2.5_ first and last-day average levels and 8-OHdG (right, edf = 1.4, p = 0.02). Models adjusted for age, BMI, physical activity, hypertension, chronic disease, SHS exposure and in the case of IL-6 time varying medication use. (Dashed lines denote 95% CIs).

No exposure time interactions were statistically significant; therefore, they were not included in the primary models for any of the biomarkers considered. The final models also included all covariates that were added *a priori* and self reported SHS exposure. Penalized spline models (Figure 3) indicated that there were no statistically significant deviations from linearity.

## Discussion

Overall our findings suggest average (calculated as the mean of two measurement days) but not daily effects of traffic related exposures on biomarkers of oxidative stress and inflammation. Specifically, we observed a statistically significant association between average PM_2.5_ levels and urine levels of the oxidative damage marker 8-OHdG. Although exposure data were not available for all work shifts during the complete work-week, this finding was consistent in direction and magnitude with reported increases of 8-OHdG associated with a 7-day moving average of ambient PM_2.5_ levels in an elderly population [15]. The same study reports an even greater increase with 21-day moving averages indicating a greater longer-term effect, and also positive effects for OC, but not for EC. In our study, effect estimates for EC and OC were also positive, but not statistically significant.

We observed a statistically significant increase in IL-6 associated with average OC, but not with other air pollutant measures. This association remained statistically significant even after controlling for other pollutants. Three and five-day moving averages of primary OC in PM exposure effects on IL-6 blood levels in elderly susceptible populations have also been reported in previous studies and appeared more pronounced than 1-day moving average exposures [41]. This would suggest a more gradual inflammatory response as compared to an acute effect. While the study populations may not be necessarily comparable, in our study, neither the effect estimates for daily exposures nor any exposure-time interactions were statistically significant, which may be a further indication that any exposure effects may accumulate over longer time windows than were assessed here. Given that this a chronically exposed population, it is possible that there is a longer lasting effect on biomarker levels and no apparent washout over the space of two days off work. Unfortunately in the present study, we lacked the data to be able to address this issue.

A number of general population studies have not shown relationships between air pollution and inflammatory markers. Specifically, studies examining PM and CRP and IL-6 levels in healthy individuals saw no significant associations [42-44]. There is evidence; however, of greater susceptibility in certain individuals such as those with coronary heart disease or diabetes [28,42]. CRP levels measured 10-14 hr after the work-shift in North Carolina State Troopers studied over 4-days, were associated with PM_2.5_ levels in their patrol cars [45], but to our knowledge those results have not been reproduced. Overall findings on the association between CRP and PM exposures in particular have been mixed with a number of studies, including randomized crossover trials, reporting no significant associations between PM concentrations and CRP levels [46].

Our results concerning endothelial activation (ICAM-1 and VCAM-1) did not yield any statistically significant findings. Modest increases associated with ambient PM_2.5_ have been reported for these markers in an elderly population, again more pronounced when 7-day or longer moving averages of exposure were considered compared to shorter periods [47]; however, our study was not designed to detect associations with averaging periods of that length. Previous studies have also shown acute increases in ICAM-1 and VCAM-1 in bronchial tissue and mucosa in healthy volunteers exposed to diesel particles in experimental settings, but at much higher exposure levels than the ones observed in this study. Furthermore, those volunteers were not chronically exposed [17,48]. Associations between VCAM-1 levels and 2-day average black carbon (BC) exposures have also been reported [49], as well as between longer BC exposure averages (4–12 weeks) and ICAM-1 [21], and lastly 1–2 day lagged associations between EC and ICAM-1 in coronary heart disease patients [16]. We found no evidence of associations between EC and these endothelial activation markers in our time windows.

Trucking industry and highway exposures have decreased in recent years, as a result of recent emissions regulations [50]. The low levels of exposures observed in the current study may have been a reason for the lack of additional significant findings. Another limitation of this study is that the sampling scheme limited biological samples and exposure measurements to two days in the space of one week, so exposure windows greater than one week and lagged daily exposure effects could not be examined. Our reliance on microenvironmental samples, instead of personal samplers for each worker for each shift also likely lead to increased exposure misclassification. We also lacked information on exposures prior and between the shifts sampled. Exposures throughout the work week were not available for a significant portion of the study participants so average exposures over the entire week could not be considered. Additional limitations include limited power to detect modest effect sizes given the final sample size of non-smoking participants, number of measurements, and limited variability in exposures. Biomarker levels were measured with state of the art and highly sensitive methods. Pre- and post-shift samples were collected on the same time on the two sampling days, and statistical models included terms for time allowing variation but may not entirely capture within-person variation and diurnal patterns for these markers. Some non-differential misclassification may be expected, and could further reduce power to detect associations between an outcome and the exposures. Additionally, the study population is a generally healthy working male population so the findings may not be applicable to more sensitive or susceptible populations or to females.

The main strength of the current study was the concurrent assessment of microenvironment exposures and biomarker sampling in a study population with regular exposure to freshly generated exhaust, in levels that overlap with those seen in the general population. All sampled terminals were in the same company, with identical control technologies and al trucks were less than 5 years old, thus excluding potential sources of differential exposure misclassification across terminals. Additionally, we have previously shown that our microenvironment as defined by job description and location within a trucking terminal are highly representative of measured personal exposures in this industry [51].

## Conclusion

Our findings suggest associations of a first and last workday average, but not daily occupational traffic related exposures with biomarkers of inflammation and oxidative stress. There was also evidence of different effects of separate components of PM on different biomarkers, specifically a PM_2.5_ effect on oxidative stress marker levels and an OC effect on inflammation marker IL-6 levels, indicating that different PM components may affect different biologic pathways.

## Abbreviations

EC: Elemental carbon; OC: Organic carbon; PM2.5: Particulate matter less than 2.5 μm in diameter; ICAM-1: Intercellular adhesion molecule 1; VCAM-1: Vascular cell adhesion molecule 1; IL-6: Interleukin 6; CRP: C-reactive protein; 8-OHdG: 8-Hydroxy-2′-Deoxyguanosine; IQR: Interquartile range; PM: Particulate matter; P&D: Pick-up and delivery; PM1.0: Particulate matter less than 1.0 μm in diameter; EDTA: Ethylenediaminetetraacetic acid; ELISA: Enzyme-linked immunosorbent assay; HPLC-ECD: High performance liquid chromatography with electrochemical detection; LOD: Level of detection; SHS: Second-hand smoke; BMI: Body mass index; SD: Standard deviation; GM: Geometric mean; GSD: Geometric standard deviation.

## Competing interests

The authors declare no competing financial or non-financial interests.

## Authors’ contributions

AMN was responsible for analysis and interpretation of data and writing the manuscript. JEH assisted with design, data acquisition, analysis, and manuscript preparation. JC assisted with data acquisition and interpretation of data. TJS was involved in the study design and interpretation of data. DWD assisted with analysis, data interpretation and manuscript preparation. BAC assisted with analysis and data interpretation. EG was involved in study design and manuscript preparation. FL made contributions in conception, design, analysis and drafting the manuscript. All authors read and approved the final manuscript.
